# The assessment of ongoing community-based interventions to prevent obesity: lessons learned

**DOI:** 10.1186/s12889-015-1563-2

**Published:** 2015-03-04

**Authors:** Jessica S Gubbels, Frida KS Mathisen, Oddrun Samdal, Tim Lobstein, Leonie FM Kohl, Ingrid Leversen, Jeroen Lakerveld, Stef PJ Kremers, Patricia van Assema

**Affiliations:** Department of Health Promotion, NUTRIM School of Nutrition and Translational Research in Metabolism, Maastricht University Medical Centre, PO Box 616, Maastricht, 6200 MD The Netherlands; Department of Health Promotion and Development, Faculty of Psychology, University of Bergen, PO Box 7807, N-5020 Bergen, Norway; World Obesity Federation, 12 Roger Street, London, WC1N 2JU UK; EMGO Institute for Health and Care Research, VU University Medical Center, Van der Boechorstraat 7, 1081 BT Amsterdam, The Netherlands

**Keywords:** Data collection, Questionnaires, RE-AIM, Interventions, Obesity, Nutrition, Physical activity, Prevention, Community

## Abstract

**Background:**

The assessment of real-life, community-based interventions to tackle obesity is an important step in the development of effective policies. Especially multi-level interventions have a high likely effectiveness and potential reach in counteracting the obesity epidemic. Although much can be learned from these initiatives, performing an evaluation of such interventions is challenging. The aim of the current article is to provide a descriptive overview of the data collection process and general results of an assessment of ongoing multi-level obesity prevention community interventions for adults in Europe, and the lessons learned from this effort.

**Methods:**

The data collection was divided into two main phases: a) finding the ongoing obesity prevention interventions by contacting key informants in each of the European Union countries and the European Economic Area, and searching existing databases; and b) collecting detailed information (including the reach, effectiveness, adoption, implementation and maintenance (RE-AIM)) of the selected interventions using questionnaires for informants in each of the interventions.

**Results:**

A total of 78 interventions from 24 European countries were included in the final sample. The number of identified interventions varied greatly per country. The interventions covered various implementation levels (national, regional or local) and determinants (physical, sociocultural, economic, political), mostly addressing both nutrition and physical activity behaviours.

**Conclusions:**

We found that many multi-level obesity prevention interventions among adults are currently active in Europe, although we found relatively few in Southern and Eastern Europe. Identifying interventions and obtaining detailed information proved to be a difficult, time consuming and painstaking process. We discuss some of the reasons why this might be the case and present recommendations based on our experiences. We suggest that future research uses a step-wise approach, keeping participant burden to a minimum. The use of personalised and tailored strategies is recommended, led by researchers who exercise flexibility, tact and patience during the data collection process.

**Electronic supplementary material:**

The online version of this article (doi:10.1186/s12889-015-1563-2) contains supplementary material, which is available to authorized users.

## Background

Obesity has become a global problem, with over half a billion obese adults worldwide [1]. In Europe, around 15% of the adult population is obese, with large variations between countries [2]. Obesity prevalence rates in Europe range as high as 25% and 29% of adults in the United Kingdom and Hungary, respectively [2]. Obesity is an important risk factor for various serious and chronic diseases such as cardiovascular diseases and type 2 diabetes [3]. The main causes for the development of obesity are an unhealthy diet, sedentary behaviours and low levels of physical activity [4], i.e. unhealthy energy balance-related behaviours (EBRB) [5]. These behaviours are, in turn, recognised to be largely the result of so-called obesogenic environmental influences [4,6-8], although robust evidence is still lacking [9].

In line with the ecological view on human behaviour, it is now increasingly recognised that obesity prevention should not be addressed in single-level interventions that target separate determinant levels of EBRB [10,11], as they do not profit from the synergy between determinants and levels [12]. Rather, integrated multi-level approaches are needed to counteract the obesity epidemic [10,13].

SPOTLIGHT [14] is a European Union-funded research project, aiming to provide a comprehensive overview of the factors necessary for the creation of effective and sustainable interventions to change lifestyles and prevent obesity [15]. As part of the SPOTLIGHT project, a systematic review of the scientific literature was conducted in order to provide an overview of the Reach, Effectiveness, Adoption, Implementation and Maintenance (RE-AIM) [16,17] of published multi-level obesity prevention interventions worldwide [18]. *Reach* refers to the extent to which the target population took part in the intervention. *Effectiveness* refers to the impact on the participants. *Adoption* refers to the extent to which the intervention delivery agents participated and how representative they were. *Implementation* refers to the extent to which the intervention components were delivered as intended. Finally, *Maintenance* refers to both the extent to which the intervention has become institutionalised (maintenance at the setting level), and the sustainability of intervention effects (maintenance at the individual level) [16]. The systematic review concluded that the highest potential impact can be achieved in multi-level interventions that focus on all levels from the beginning of the planning process, apply diffusion theory to guide the implementation process, and use a website to disseminate the intervention [18].

In addition to reviewing published interventions, a great deal can be learned from *ongoing* interventions. The interventions that are published are often only the larger interventions, coupled to scientific research studies designed to evaluate the intervention effects (e.g. randomised controlled trials), and often conducted by large research institutes such as universities [18]. However, many real-life health promotion and obesity prevention interventions are small-scale, community-led, local initiatives, and often practice-based rather than evidence-based. Although such interventions will presumably have less information available on the RE-AIM characteristics, the information that is available has not been systematically evaluated to date, and such interventions are not likely to be included in reviews of ‘what works’. A separate work package within SPOTLIGHT therefore aimed to provide an overview of the general characteristics and RE-AIM characteristics of ongoing multi-level interventions in Europe, which target overweight/obesity through changes in nutrition, sedentary and physical activity behaviours among (subgroups) of adults in a community [15]. The results of this survey have been collated in the form of an interactive, freely available Webatlas [19]. The detailed findings describing the RE-AIM assessments of these interventions will be published separately (paper in preparation).

The aim of the current article is to describe the process and general results of the search for these types of unpublished ongoing multi-level obesity prevention interventions, and the lessons learned from this search, to guide future research efforts. We will first discuss the definitions used in the current study, after which we describe the process of the data collection. In addition, we will describe some general results with regard to the included interventions, and finally we will discuss our conclusions and the implications for research and practice.

## Methods

### Definitions

The aim of the study was to provide an overview of ongoing multi-level interventions in Europe which aim to influence overweight/obesity-related nutrition, sedentary and physical activity behaviours among (subgroups of) adults in a community. Before the data collection could be started, several definitions had to be clarified.

First of all, the interventions had to be *ongoing*. For the purpose of this study that meant the inclusion of any intervention that was actively running in at least part of 2012, the year in which the data collection started. This included interventions that had stopped during the data collection period, interventions that were expected to stop in the coming few years, and interventions that had only just started.

Second, we were looking for *interventions.* We took a broad perspective on this term and included all kinds of programs, projects, approaches and initiatives. This included for instance mass media campaigns, but also small-scale local bottom-up community initiatives. The nature of the organization which was developing, funding or implementing the intervention was not a selection criterion: interventions from all kinds of organizations were included (including research led initiatives, community led initiatives, and research-community partnerships).

Third, the interventions had to be *multi-level* interventions, which was defined as targeting both the individual *and* environmental determinants of EBRBs [15]. These environmental determinants could be at a single environmental level or type, but also at multiple environmental levels or types. For example, many interventions targeting obesity include some kind of group meetings, such as educational sessions, workshops or group sports lessons [18]. These meetings are intended to target individual determinants of EBRB, such as knowledge and skills [18]. However, without specifically intending to target this, these meetings could also influence the social environment of the participants, as they might receive social support from the other participants or the motivating atmosphere created in such a group [20].

Fourth, the interventions had to address *obesity prevention.* While many lifestyle behaviours have been linked to obesity, we chose to include only interventions targeting behaviors that have been linked to obesity with at least *probable* evidence. For nutrition this included sugar-sweetened beverages intake, energy-dense food intake, dietary fiber intake (protective), breakfast and meal patterns and snacking behavior. For physical activity this included sports and exercise, leisure time physical activity, active transport, occupational physical activity and household activities. And finally for sedentary behaviour, this included sitting, television viewing, reading and computer time [15,21].

Obesity treatment was explicitly excluded in the current study. However, as the treatment of *overweight* can be regarded as a prevention of *obesity*, we included overweight treatment, as long as the treatment was focussed on changing EBRB, thus excluding clinical treatment. Obese individuals could be part of the target population, but not the sole target population.

Fifth, the included interventions had to be *community* interventions. We decided to apply a broad definition, and defined a community as a group of people mutually connected by the geographical area of habitation (e.g. neighbourhoods, cities, regions, nations).

Finally, the included interventions had to be implemented in Europe. For practical purposes we limited this to the EU member states plus the European Economic Area countries (Iceland, Norway and Switzerland). Interventions could be implemented at a national level, regional level (in part of a country), or local level (in a municipality, city, suburb or village).

### Data collection

Data were collected by four of the authors (JSG, FKSM, LFMK and IL), who were university researchers at the time of the data collection. All data were recorded using Microsoft Excel 2010.

The data collection was conducted between September 2012 and March 2014, and was split into two main phases: a first phase to identify relevant interventions, and a second phase to collect detailed and accurate information on each of the identified interventions. An overview of the phases and steps can be found in Table 1. This phased approach is similar to the procedure used in a previous EU-funded study that made an inventory of community-based initiatives to combat childhood obesity [22].

**Table 1:** **Overview of data collection phases, steps, sources and instruments**

**Phase**	**Step**	**Sources/Instruments**
1: Identifying interventions	1: Searching existing sources	Sources: Databases, abstract books, internet
	2: Identifying and approaching key informants	Sources: Researchers’ professional networks, EU projects, public health networks, government departments, non-governmental organisations. Instrument: Short questionnaire (Additional file 1)
	3: Identifying contact persons of interventions, collecting general information on interventions	Sources: Key informants, internet
2: Collecting and verifying detailed information	1: Selecting interventions	Instrument: Research team analysis of short questionnaire and other available information
	2: Collecting detailed information on interventions	Instrument: Partially pre-filled extensive questionnaire (Additional file 1)

The first phase was further split into several steps (see Table 1). First, various sources were searched for suitable interventions. This included searching existing intervention databases, such as the online intervention databases of the Dutch Centre for Healthy Living [23], which provides an overview of interventions currently available in the Netherlands, and the level of evidence supporting these interventions. In addition, reports on interventions (e.g. [24-26]), recent abstract books of relevant conferences (e.g. the European Congress on Obesity) and the internet were searched for suitable interventions. Search terms were combinations of ‘intervention’ or synonyms for this term (e.g. program, project, approach, initiative) and ‘obesity’ or related terms (e.g. BMI, overweight, lifestyle, health, nutrition, diet, physical activity, exercise). In a second step we searched for key informants in the area of obesity prevention, identified through the researchers’ professional networks, EU projects, public health networks, government departments and non-governmental organisations. These key informants were then asked whether they knew of any relevant interventions or could lead us to other key informants. People were approached on a personal level where possible. To minimise the amount of work that had to be put in by respondents, the emails and questionnaire sent out to key informants in phase 1 were very short. The template used for the email can be found in Additional file 1. The third step was to identify contact persons for all relevant interventions. These might be project leaders, researchers, employees of health organisations, municipalities or governments, or any other person involved with the interventions. We then collected general information regarding the interventions (in order to assess whether the interventions fulfilled the selection criteria) from these contact persons via email, phone, or in person, as well as from the earlier identified information sources (e.g. websites, reports, key informants). For some interventions contact persons could not be identified, in which cases we collected the information from the original information sources. From this information, we selected only the interventions fitting the inclusion criteria.

In the second main phase (see Table 1), for feasibility reasons, we selected a representative sample of interventions out of all relevant interventions identified. The selected interventions reflected all types of interventions encountered per country. This means that one example intervention was included for each intervention type encountered, with a maximum of 10 interventions per country. For instance, if there were multiple interventions within one country combining physical activity group sessions with information meetings, we would select only one of these interventions as an example. We then collected detailed information regarding the selected interventions, using an extensive questionnaire (see Additional file 1) to gather general information and information on the intervention’s RE-AIM characteristics. This assessment was based on earlier studies [16,17,27]. To minimise the amount of work that had to be put in by respondents, we completed all parts of the questionnaire for which information was already available from other sources. Respondents then merely had to correct any errors and fill in the ‘gaps’.

In all phases and steps, repeated and personalised reminders were used. Mail, e-mail and phone were used, depending on the contact information available, as well as the preferences of the respondent. In addition to these personalised approaches and reminders, all correspondence emphasised the importance of participation (supporting obesity prevention), as well as the benefits for the respondent (e.g. publicity for their intervention through publication in the online WebAtlas [19]).

## Results

In the first phase of the data collection we identified a total of 211 potentially eligible interventions from 24 countries. The number of interventions per country ranged from 0 to 45. A total of 78 interventions from the 24 countries were selected for detailed information collection in the second phase of the data collection. Table 2 shows details of the 78 interventions. Detailed information regarding the RE-AIM of each of the interventions can be found in the Webatlas [19].

**Table 2:** **Ongoing multi-level obesity prevention interventions, included in the SPOTLIGHT project**

**Country**	**N selected inter-ventions**	**Level**			**Type of multi-level intervention** ^**a**^			**Target behaviors**				**Target populations**						**Funding source**		
		***Natio-nal***	***Regio-nal***	***Local***	***Ind. + SC env.***	***Ind. + per-ceived phys env.***	***Ind. + multiple env. types***	***Nutri-tion***	***PA***	***Nutri-tion and PA***	***Lifestyle or health in general*** ^***b***^	***Gener-al (adult) popu-lation***	***Over-weight***	***Low SES***	***Seniors***	***Ethnic mino-rities***	***Other sub-groups*** ^***c***^	***Govern-mental and NGO’s*** ^***f***^	***For-profit organi-sations*** ^***g***^	***Partici-pants***
*Austria*	**3**	2	0	1	0	0	3	1	0	3	0	1	1	1^e^	1^e^	0	0	3^h^	0	1^h^
*Belgium*	**6**	0	4	2	3	0	3	2	3	1	0	2	0	3^e^	1	1^e^	0	6	0	0
*Bulgaria*	**1**	1	0	0	0	0	1	0	0	1	0	1	0	0	0	0	0	1	0	0
*Cyprus*	**0**	-	-	-	-	-	-	-	-	-	-	-	-	-	-	-	-	-	-	-
*Czech Republic*	**1**	1	0	0	^d^	^d^	^d^	0	0	0	1	1	0	0	0	0	0	1	0	0
*Denmark*	**6**	1	0	5	3	0	3	1	1	2	2	4	1	0	1	0	0	5	1	0
*Estonia*	**2**	1	1	0	1	0	0	1	1	0	0	1	0	0	0	0	1	2	0	0
*Finland*	**4**	2	2	0	2	0	2	1	1	1	1	3	0	0	0	0	1	4	0	0
*France*	**3**	2	1	0	^d^	^d^	2^d^	0	0	2	1	2	0	1	0	0	0	2	1	0
*Germany*	**4**	2	2	0	2	0	2	0	1	0	3	1	0	0	2	1	0	4	0	0
*Greece*	**0**	-	-	-	-	-	-	-	-	-	-	-	-	-	-	-	-	-	-	-
*Hungary*	**1**	1	0	0	0	0	1	0	1	0	0	0	0	0	1	0	0	^i^	^i^	^i^
*Iceland*	**2**	2	0	0	2	0	0	0	2	0	0	1	0	0	0	0	1	2	0	0
*Ireland*	**4**	2	1	1	2	0	2	1	1	0	2	3	0	0	0	0	1	4^h^	1^h^	1^h^
*Italy*	**0**	-	-	-	-	-	-	-	-	-	-	-	-	-	-	-	-	-	-	-
*Latvia*	**4**	0	1	3	1	0	3	1	3	0	0	2	0	0	2	0	0	4	0	0
*Lithuania*	**0**	-	-	-	-	-	-	-	-	-	-	-	-	-	-	-	-	-	-	-
*Luxem-bourg*	**1**	1	0	0	0	0	1	0	0	1	0	1	0	0	0	0	0	1	0	0
*Malta*	**2**	1	0	1	0	0	2	1	0	0	1	2	0	0	0	0	0	2	0	0
*Nether-lands*	**10**	1	0	9	1	1	8	0	3	2	5	5	0	1^e^	2^e^	2^e^	2^e^	10^h^	4^h^	0
*Norway*	**8**	2	3	3	1	0	7	1	3	2	2	5	1	0	0	1^e^	2^e^	7	1	0
*Poland*	**1**	1	0	0	0	0	1	0	0	1	0	1	0	0	0	0	0	1	0	0
*Portugal*	**0**	-	-	-	-	-	-	-	-	-	-	-	-	-	-	-	-	-	-	-
*Romania*	**2**	2	0	0	^d^	^d^	^d^	0	0	2	0	2	0	0	0	0	0	2	0	0
*Slovakia*	**1**	1	0	0	0	0	1	0	1	0	0	1	0	0	0	0	0	1	0	0
*Slovenia*	**1**	0	1	0	^d^	^d^	^d^	0	0	0	1	1	0	0	0	0	0	1	0	0
*Spain*	**3**	2	1	0	1	0	2	1	0	1	1	2	0	0	0	0	1	3^h^	1^h^	0
*Sweden*	**2**	1	1	0	0	0	2	0	0	2	0	2	0	0	0	0	0	2	0	0
*Switser-land*	**2**	1	1	0	0	0	2	0	2	0	0	2	0	0	0	0	0	2^h^	1^h^	0
*United Kingdom*	**4**	1	2	1	1	0	3	0	1	0	3	2	1^e^	0	1^e^	0	2^e^	4^h^	1^h^	0
***Total***	**78**	**31**	**21**	**26**	**20** ^**d**^	**1** ^**d**^	**51** ^**d**^	**11**	**23**	**21**	**23**	**48**	**4** ^**e**^	**6** ^**e**^	**11** ^**e**^	**5** ^**e**^	**9** ^**e**^	**74** ^**h**^	**10** ^**h**^	**2** ^**h**^

*SC = socio-cultural; SES = socio-economic status; env. = environment; phys. = physical; PA = physical activity.*

^*a*^ *Levels of determinants targeted. There were no interventions targeting individual level determinants of behaviour in combination with solely physical environment, economic environment or political environment.*

^*b*^ *E.g. also focussing on alcohol, smoking, stress, mental health, sleeping or other health-related behaviors.*

^*c*^ *E.g. men or women, employees, pregnant women.*

^*d*^ *No details regarding the multi-level type of one or several interventions known.*

^*e*^ *One or more interventions focussing on multiple specific target groups or combinations of target groups.*

^*f*^ *Including the EU and universities.*

^*g*^ *Including employers.*

^*h*^ *One or more interventions funded by funders from multiple categories.*

^*i*^ *No details regarding funding for one or several interventions known.*

Of the 78 selected interventions, 31 were national interventions, 21 were regional interventions, and 26 were local interventions. It can be seen that, in countries where few (one or two) interventions were found (e.g., Bulgaria, Czech Republic, Hungary, Luxembourg, Slovakia), they were most often national or regional interventions, such as mass media campaigns. By contrast, where more than five interventions were found, they were most often local or regional interventions (see Table 2). This raises the question whether there are indeed fewer local and regional interventions in certain countries, or whether these projects were not identified using our search methods.

The majority of the included interventions (51 of the 78) addressed determinants across several *types* of environments (i.e. physical, sociocultural, economic, or political) in addition to individual determinants of EBRB (see Table 2). In addition, all interventions but one [28] were found to include a socio-cultural component. The way the socio-cultural environment was targeted varied greatly: at the micro level, several interventions included group meetings such as group physical activity sessions in the ‘Bewegen op Recept’ (Exercise on Prescription) intervention from Belgium, or informational group sessions on healthy eating such as those in the ‘Frisklivssentralen i Modum’ (Healthy Lifestyle Centre in Modum) intervention from Norway. At the macro level, intervention components targeting the socio-cultural environment included mass media campaigns such as the ‘Lífshlaupið’ (Life Run) intervention from Iceland, promoting participation in a competition to be active.

The majority of the interventions targeted either physical activity, a combination of physical activity and nutrition, or lifestyle in a broader sense (including components focussing on, for instance, smoking, stress or alcohol consumption; see Table 2). Nutrition behaviour as a sole target was registered the least often (11 interventions). An example of such an intervention was the ‘Healthy food made easy’ intervention from Ireland, which comprised nutrition and cooking courses in groups. An intervention focussing solely on physical activity was the ‘Veloland Schweiz’ (Cycling in Switzerland) intervention, promoting leisure cycling using a website and a mobile application (‘app’) with routes and information, restructuring of the cycling infrastructure and cycling events. An example of an intervention focussing on both nutrition and physical activity was the ‘Schlank ohne Diät’ (Slim without dieting) intervention from Austria, in which exercise sessions were combined with informational group sessions on healthy nutrition and physical activity. An example of an intervention focussing on lifestyle in general was the ‘Sundhetscafé i et socialt boligområde’, a Community Health Café in which inhabitants of Roskilde (Denmark) could get free health check-ups, join group exercise sessions, and also receive counselling from professionals about smoking cessation, healthy nutrition and physical activity.

Over half of the included interventions targeted the general population or the general adult population. Far fewer interventions targeted a specific group, such as seniors or elderly (11 interventions), low socio-economic status (SES) groups (6 interventions) or ethnic minorities (5 interventions; see Table 2).

Most interventions were partly or fully funded by governmental organisations or NGOs (74 interventions). Ten interventions were funded or co-funded by for-profit organisations. An example of an intervention co-funded by a for-profit organisation was the Danish Whole Grain Campaign (‘Fuldkornskampagnen), which originated from a public-private partnership between various commercial organisations in the food industry and NGOs. Some interventions were partly funded by the participants, who were asked for a financial contribution (see Table 2).

## Discussion

This section discusses the lessons we learned from the search for ongoing multi-level obesity prevention interventions for the SPOTLIGHT project [15].

The number of interventions found was limited. There are several explanations for this. First of all, there simply are relatively few multi-level obesity prevention interventions. In the systematic review of multi-level interventions published in peer-reviewed journals [18], only seven were identified in Europe. Many of the interventions addressing obesity that were initially identified during the current data collection process either used a single component approach (e.g. solely adjusting the environment or only targeting individual level determinants) instead of a multi-level approach; or they focussed on children and adolescents, or targeted people already suffering from obesity (i.e. applying treatment instead of prevention). An additional reason for the low number of interventions found is that there were few *ongoing* interventions. An problem with interventions that are no longer active is that the contact persons cannot always be reached. Several interventions stopped just before or during the data collection. In addition, few interventions were long-term; they are often implemented for a fixed time period of a few years. This was also found in the systematic review of multi-level interventions [18], which found low levels of sustainability and dissemination of interventions.

An alternative explanation for the low number of interventions is that our search methods were inadequate, and we failed to find them. We encountered various problems during the data collection that might result in missing out on interventions. However, given the variety of data collection methods used, and the efforts made to identify interventions, we believe the examples reported are representative of the interventions being undertaken.

An uneven distribution between countries regarding the number of interventions was observed. This might be attributed to a bias introduced by the researcher and methods, but we think it is probable that there are actual differences between countries, for various reasons. Fewer obesity prevention interventions were identified in the Eastern (i.e. Bulgaria, Czech Republic, Hungary, Poland, Romania, Slovakia, Slovenia) and Southern (i.e. Cyprus, Greece, Italy, Portugal, Spain) European countries. This is in line with the systematic review of published interventions within the SPOTLIGHT project: the seven multi-level interventions identified in Europe were all from Northern and Western European countries (Norway, Belgium, the Netherlands, and the UK) [18]. Also a previous study on ongoing European community interventions against childhood obesity showed fewer interventions in Southern and Eastern Europe, with the exception of Spain [22]. Such regional differences could be attributed to health promotion itself receiving less priority in some countries, or being the victim of economic cut-backs: although cuts in budgets for public health and health promotion have been noticed throughout Europe [29], they may be more severe in some countries, such as Greece [30].

In line with previous reporting [18], the majority of the interventions focused on a combination of both physical activity and nutrition. EBRBs are known to often co-occur (so-called clustering), with inactive individuals often also consuming unhealthy diets [31]. By addressing physical activity and nutrition simultaneously, the synergy between clustered behaviours can be utilised, which is hypothesised to maximise intervention results [32]. In line with the SPOTLIGHT systematic review [18], the large majority of the interventions further targeted multiple types of environmental determinants of EBRB in addition to individual determinants. The socio-cultural environment was addressed as part of almost all of our 78 interventions, which is also in line with the systematic review [18].

An important feature of the data collection was the diversity of ways in which the data was collected. Each country required a different approach. For instance, for Luxembourg, a relatively small country, the researchers went to the Ministry of Health for a face-to-face interview. For larger countries, communication mostly went via email or telephone. There were also differences in the data collection between interventions within each country, depending on the contact information available for each intervention, and the preferences of the informants and respondents.

Several of the data collection approaches were successful for the identification of relevant interventions and information regarding the interventions: Using a stepwise approach, keeping response burden low, and using tailored and personal approaches. The stepwise approach ensured feasibility and made the data collection manageable, not only for the researchers, but also for the respondents. By asking them information in small ‘portions’, they were less likely to drop out during the data collection process. In line with this, the amount of work that had to be put in by respondents was minimalized, using short communications and utilising any information that had been collected previously. Previous research among health care professionals (physicians) has also shown the importance of a low research burden, shifting as much of the work as possible away from the participants to the research staff [33]. The phased data collection procedure in the current study is similar to the procedure used in a previous study that made an inventory of community-based initiatives to prevent childhood obesity [22]. In that study, the researchers also started by asking key informants and searching databases for interventions and contact persons, after which in a second step they collected detailed information from the contact persons. Another strategy that worked well was to use personal and tailored approaches when contacting respondents. Personal contact is one of the known success factors for recruiting health care professionals for research studies [33]. Tailored communications are more likely to be read and to be perceived as relevant [34]. A disadvantage of the personal approach using the researchers’ own professional networks was that the results might have been influenced by the researchers: a larger network might result in more contacts than a smaller network, and the researchers’ network is likely to be centred in the field of research and geographical region in which the researcher is working. However, the researchers also called upon respondents to spread the calls for interventions and key informants to their own networks. Such peer-to-peer recruitment has previously been indicated as a successful strategy for recruiting health care professionals for research [35].

There were also several strategies that were less successful. In the first phase of the data collection the researchers used mass emailing to approach possible key informants. Several problems were encountered with this approach, the first being who should be contacted. Key informants and contact persons frequently changed during the data collection, and for some interventions or organisations contact persons or contact information for these persons could not be found. The second problem with mass emailing was the very low response. The same problem arose when contacting networks of professionals, as described in the methods section. To illustrate, at some point a mass emailing was sent out to a total of 313 possible contact persons. A follow-up message was also sent. Out of those 313, only 11 people responded (3.5%).

Approaching people through existing networks in the current study often provided a response rate that was even lower. A major challenge is therefore to build motivation and commitment from professionals in the public health field to contribute to the development of a better evidence base to evaluate ongoing practice. Leaders in public health should take responsibility to establish such commitment.

Another approach which met difficulty was searching the internet. Search terms requiring the term ‘intervention’ and ‘obesity’ could easily miss important interventions, as they might be called programs, projects, approaches, initiatives or any other alternative for ‘intervention’, and might use terms such as lifestyle, health, nutrition, (physical) activity and well-being in their title, rather than obesity. A second problem with searching the internet was that the information found online was mostly outdated, as websites are often not regularly updated or reviewed by the search engines.

A further problem with using the internet to find interventions was that many interventions do not have their own website, or are not referred to on other websites. This is especially the case for the more local, small-scale interventions. Similar problems were encountered when using reports, databases and existing overviews of interventions. Such reports are quickly outdated, and did not result in the identification of many *ongoing* interventions. Moreover, these overviews often only list interventions that are already published or in some way connected to research, again excluding the small-scale interventions. However, although the internet and existing databases and reports were not successful approaches for finding interventions, they were often helpful for finding detailed information regarding some of the interventions that were already identified.

There were also some language issues. Most communication took place in English, with the exception of some communication in Dutch and Norwegian/Swedish (i.e. the mother tongue of the researchers). There was no budget to hire translators. The extent to which this resulted in problems differed between countries. Although English is by far the most commonly used language in Europe [36], it severely impeded the communication with the contact persons and key informants in some countries. For instance in France and the French speaking part of Belgium, communication in English was often problematic, with a low response rate, or responses being sent in French. Research has shown large differences in the English skills in the different European countries, ranging from about a quarter of citizens being able to have a conversation in English in Hungary, the Czech Republic, Slovakia, Spain and Portugal, up to almost nine out of ten in the Netherlands, Malta, Sweden and Denmark [36].

Gathering information on the RE-AIM characteristics also posed particular problems. The interventions identified in the current study were often not coupled to scientific research, and the people working with the interventions often did not have an academic background. We do not want to want to imply that interventions should always originate from an academic background. Instead we would recommend having academics *support* community led intervention development. A good example of this is ‘Woerden Actief’ (Woerden Active), an intervention which was initiated by a group of residents of Woerden (a municipality in the Netherlands) as a civic initiative. Later on, researchers got involved through the Municipal Health Service and other professional organisations. To date the intervention is still ongoing and successful [19].

Parallel to the involvement of researchers in community led initiatives, we also need to look at the other side of the spectrum. More focus needs to be on the implementation and dissemination of researcher led interventions. Lately, attention to these issues has been increasing, for instance by requiring researchers to outline their plans for dissemination and continuation when first applying for funding. Especially the involvement of linkage systems with representatives from the community throughout the intervention development and implementation is recommended (e.g. [37]).

Information was difficult to extract, with respondents saying they had insufficient time to respond to the questionnaires and emails or that they were not motivated to respond as they were not interested in publicity for their intervention, or that they did not perceive the relevance of participating in the study. These reasons for non-response were most often mentioned when little information from other sources could be pre-filled into the questionnaire and more information was thus requested from the respondent, increasing the respondent burden. Other respondents indicated that they did not feel competent to provide the needed information, or implied that they feared criticism or shame for not being able to provide certain information. Some of them further felt the questionnaires were like an inspection, in which the respondents were being graded or examined. These feelings of respondents should be taken into account in future studies to optimise response rates. The aims and use of the reported data should be made clear to the respondents. Another problem was that certain information regarding interventions was confidential, because of the involvement of commercial organisations that owned copyright of the intervention process or materials. In other cases, the information was confidential because the research studies coupled to the interventions had not been published yet. An embargo on the data until a certain date could perhaps help in such cases.

There are some other data collection methods which could have been used and which we would like to recommend for future projects, but which were not considered feasible within the current project. Local researchers could have been employed for the data collection in each specific country. This would resolve the cultural and language barriers that we faced in the current study. In addition, such local researchers could use their own national networks for the data collection, as the researchers did in the current study for the Scandinavian countries (for the Norwegian members of the research team), the Netherlands and Belgium (for the Dutch team members) and the UK (for the English team members). We therefore recommend funding for local researchers or translators to be taken into account in multi-country studies, to ensure a better response rate.

As a result of these factors, the completeness of the intervention descriptions was low. Information was especially lacking for smaller and local interventions. In particular, information regarding the RE-AIM characteristics of interventions was often lacking, which has also been noted for interventions with a scientific background [18]. More information on the external validity and sustainability of interventions is needed in order to take informed decisions about intervention development and implementation.

## Conclusions

Identifying ongoing interventions and obtaining detailed information on such interventions is a difficult, time consuming and painstaking process. One of the difficulties in collecting data may lie in the priorities of informants, with many of them unwilling or unable to make the necessary time available for answering a questionnaire. We urge all professionals in the public health sector to appreciate the importance of sharing their experiences of health promotion interventions, in order to improve the evidence base for effective policy-making. By publishing the information shared for the current review in an online, freely available database of ongoing interventions in the EU [19], we want to set an example for interventions worldwide to share their experiences, but also make optimal use of what is already available. Furthermore, strong leadership in the public health field might play an important role in establishing a commitment to sharing information and experiences. In addition, in line with the World Health Organisation recommendation to allocate a minimum of 10% of the total financial resources for a health promotion initiative to evaluation [38], we recommend devoting resources to the evaluation of *all* interventions initiatives taken.

Future research can utilise our experiences. We recommend that data collection methods use a stepped approach, the use of personalised and tailored strategies, communication in the mother tongue of respondents, making efforts to minimise participant burden and in particular being flexible during the data collection process.

As regards the results of our search, there seems to be large variety of ongoing multi-level interventions preventing obesity among adults in the EU, although there are relatively few interventions in certain regions. This is striking, given the urgency of the current obesity epidemic and the likely effectiveness [10-13] and potential reach [18] of multi-level interventions in counteracting this epidemic.

## Additional file

Additional file 1:
**Email message used for the identification of key informants and ongoing multi-level obesity prevention interventions (first phase data collection); and questionnaire used for the collection of detailed information (second phase data collection) regarding ongoing multi-level obesity prevention interventions for the SPOTLIGHT project.**

## Abbreviations

EBRB: Energy balance-related behaviour
EU: European Union
PA: Physical activity
RE-AIM: Reach, efficacy, adoption, implementation and maintenance
SPOTLIGHT: Sustainable prevention of obesity through integrated strategies
UK: United Kingdom
