# Supplementary material for: The assessment of ongoing community-based interventions to prevent obesity: lessons learned
Source: BMC Public Health. 2015 Mar 4;15:216. doi: 10.1186/s12889-015-1563-2 (PMC4351935; doi:10.1186/s12889-015-1563-2)
Supplement: Additional file 1: — Email message used for the identification of key informants and ongoing multi-level obesity prevention interventions (first phase data collection); and questionnaire used for the collection of detailed information (second phase data collection) regarding ongoing multi-level obesity prevention interventions for the SPOTLIGHT project. [file 12889_2015_1563_MOESM1_ESM.docx]

**Appendix 1: Email message used for the identification of key informants and ongoing multi-level obesity prevention interventions for the SPOTLIGHT project (first phase data collection)**

**SPOTLIGHT – OBESITY PREVENTION IN ADULTS**

Dear [respondent],

SPOTLIGHT is a cross-European research project and stands for sustainable prevention of obesity through integrated strategies. It is funded by the European Commission through its seventh Framework programme.

As part of the project we are going to collect and present online through a WebAtlas technology information about **ongoing** **multi-level** **interventions** **preventing obesity** among **adults** in all **EU-countries**. Examples of included interventions are an intervention creating physical activity opportunities in local neighborhoods as well as pointing out these opportunities to local inhabitants; and an intervention referring individuals at high risk for obesity to physical activity group sessions or educational meetings. The scope of interventions could be physical activity, nutrition and/or sedentary behaviours. It may be community based projects as well as more large-scale national projects.

We ask your help with the following:

1. Do you personally know such obesity prevention intervention(s) in your country? If yes, where (websites, reports) or from whom (contact persons) can we get more information about the intervention(s)?
2. Who may we contact to get information about such interventions aimed at adults in your country? Please, provide name and contact details (email and/or telephone).

Please also let us know if you do not know any relevant contact persons or interventions. Thank you very much for your help!

[sender]

[affiliation sender]

Please send your response to: **[email address]**

**Appendix 2: Questionnaire used for the collection of detailed information (second phase data collection) regarding ongoing multi-level obesity prevention interventions for the SPOTLIGHT project**

**SPOTLIGHT Questionnaire**

**[TITLE OF INTERVENTION** *English***]**

**([Original title])**

This is a questionnaire to collect information on multi-level interventions for the Spotlight project. This questionnaire is divided in two parts ‘General characteristics’ and ‘RE-AIM’. The bold text form questions under which answers can be filled out. Questions that are filled out in advance or partially need to be verified. Thank you for participating!

*General characteristics*

- **Give a general description/summary of the intervention**
- **Give a description of the intervention components**

*For level, choose one or more of the following levels this intervention component targets at:*

*Individual level (e.g., motivational factors, self-regulatory skills, perceived environment)*

*Micro physical level (e.g., sports facilities, walkability, vending machines)*

*Micro socio-cultural level (e.g., family, colleagues, social support)*

*Micro economic level (e.g., costs of sports activities and foods)*

*Micro political level (e.g., workplace policies)*

*Macro physical level (e.g., structural neighbourhood planning)*

*Macro socio-cultural level (e.g., mass media campaigns)
Macro economic level (e.g., subsidies on fruit or sports)
Macro political level (e.g., regional or national laws)*

1. Intervention component 1

*Level*:

*Content*:

1. Intervention component 2

*Level*:

*Content*:

1. Intervention component 3

*Level*:

*Content*:

1. Intervention component 4

*Level*:

*Content*:

1. Intervention component 5

*Level*:

*Content*:

1. Intervention component 6

*Level*:

*Content*:

1. Intervention component 7

*Level*:

*Content*:

1. Intervention component 8

*Level*:

*Content*:

- *In what type of community is the intervention conducted*
- **In which country is the intervention conducted?**
- **In which area(s) within this country is the intervention conducted? (e.g. which cities, regions)**

- **How many people live in this area(s)?**
- **Does this area have any specific characteristics which could be important for the intervention? (e.g., overrepresentation of certain ethnic groups, specific socio-economic characteristics)**
- *Initiators of the intervention*
- **Is the intervention initiated by a certain organisation? What kind of organisation is this initiating organization?**
- **Did this organization also develop this intervention? If not, who developed the intervention?**
- *Time frame of the intervention*
- **When was the intervention developed?**
- **When was the intervention implemented?**
- **Is the intervention still on-going?**
- **If the intervention is still on-going, until when is it (at least) planned to run?**
- *Use of theory*
- **Was the intervention developed using a certain theory?**
- **Was the intervention spread/implemented using a certain theory?**
- *At what specific behavioural changes is the intervention targeted*
- **Is the intervention targeted at nutrition? If yes, at which specific nutrition-related behaviours is it targeted? (e.g., fruit intake, breakfast consumption, snack intake)**
- **Is the intervention targeted at physical activity? If yes, at which specific physical activity-related behaviours is it targeted (i.e. which type and/or intensity)? (e.g., active transport, sports, moderate to vigorous physical activity)**
- **Is the intervention targeted at sedentary behaviour? If yes, at which specific sedentary behaviours is it targeted? (e.g., television viewing, computer use)**
- **Are there any other behaviours that are targeted in the intervention? (e.g., smoking, alcohol consumption, stress)**
- *Aims of the intervention*
- **What were the specific aims of the intervention in terms of behavioural change (nutrition, physical activity, sedentary behaviour)? How much do the targeted behaviours need to change? (e.g., reaching a certain norm, accumulating a certain amount of activity, lowering intake of a certain item by xx%)**
- **What were the specific aims of the intervention in terms of BMI or overweight reduction/prevention? (e.g., to lower or halt overweight prevalence, maintain or decrease average BMI, and by how much)**
- **Where there any other aims of the intervention? (e.g. reduction of blood pressure, mental complaints)**
- **When do these aims have to be reached?**
- **Are there different aims for different sub groups? (e.g., younger vs. older participants, men vs. women, different ethnic groups)?**
- **To what extent did the intervention take into account socioeconomic inequalities?**
- **Has a needs assessment been done to develop the intervention?**
- *How is the intervention funded*
- **How was the intervention development funded?**
- **How was the implementation of the intervention funded?**
- **How is the continuation of the intervention funded?**

*RE-AIM*

*Reach*

- *Who is targeted with the intervention*
  - **What is the targeted geographical area? (e.g., a certain city or region?)**
  - **How many people are targeted by the intervention?**
  - **What is the age range of the target population?**
  - **Are both males and females targeted? If yes, what is the proportion of both?**
  - **Is the intervention aimed specifically at subjects with specific characteristics with regard to for instance socio-economic status, employment, demographics?**
  - **Is the intervention aimed specifically at subjects with a specific disorder or at high risk for such disorders? (e.g., diabetes, cardiovascular diseases, etc)**
  - **Is the intervention aimed specifically at overweight/obese subjects? If not, what is the proportion over overweight/obesity in the target population?**
  - **Are there any other specific inclusion criteria?**
  - **Are there exclusion criteria?**
- *To what extent is the targeted group reached*
  - **How many people are actually exposed to / participate in the intervention?**
  - **Do those reached live in a certain subarea within the targeted area or is the whole target area reached?**
  - **What is the age range of those reached?**
  - **What is the proportion of males and females among those reached?**
  - **Do those reached have specific characteristics with regard to e.g. socio-economic status, employment, demographics?**
  - **Do those reached have specific disorders or are they at high risk for such disorders? (e.g. diabetes, cardiovascular diseases)**
  - **What proportion of those reached is overweight/obese?**
  - **Do those reached have any other specific characteristics?**
- *Who is reached by what combination of intervention components*
  - **Are those reached, reached by all components? If not, please indicate per component how many people are reached, as well as any specific characteristics that vary between those reached of the components.**

*Efficacy*

- *What are the effects of the components and the intervention as a whole*
  - **Are there changes in the physical activity of those reached?**
  - **Are there changes in the diet of those reached?**
  - **Are there changes in sedentary behaviour of those reached?**
  - **Are there changes in the body mass index or percentage of overweight/obese?**
  - **Are there any other biological changes, such as lower blood pressure?**
  - **Are there psychological changes, such as lower stress levels, improved mental health?**
  - **Are the effects of the intervention different in different sub groups? (e.g. younger vs. older participants, men vs. women, different ethnic groups)**
- *To what extent were the targeted outcomes achieved*
  - **What are the target outcomes of the intervention?**
  - **Are the target outcomes achieved?**
- *Where there any adverse effects*
  - **Are there any negative consequences or side effects due to the intervention (e.g. sports injuries, etc.)?**
- *Partners*
  - **Which organizations were involved in the evaluation of the intervention?**

*Adoption*

- *Which community organizations can deliver the intervention*
  - **Which and how many organizations are needed to deliver the intervention?**
  - **Do the delivering organizations have to have certain characteristics, goals or activities?**
  - **Do the delivering organizations have to be located in a certain area?**

*Implementation*

- *Involvement participants*
  - **Where participants actively involved in the design and/or implementation of the intervention?**
- *To what extent do interventionists have the needed expertise and training*
  - **Do the people delivering the intervention need certain expertise?**
  - **Is there a training to ascertain this expertise?**
  - **How many of the people delivering the intervention have followed the training?**
  - **What are the costs of the training?**
- *To what extent do expected/needed organizations participate in the intervention*
  - **Are there any organizations not (yet) participating that should (preferably) participate?**
- *What is the intervention’s completeness of implementation*
  - **To what extent did the participants receive the intervention components?**
- *What is the intervention’s fidelity of implementation*
  - **Is the intervention delivered as intended? Are there any modifications? If yes, what are these modifications?**
- *Adapted to local circumstances.*
  - **Was the intervention based on a previous intervention?**
  - **If yes, is the intervention adapted to local circumstances and how?**
  - **Did this increase the efficacy of the intervention?**

*Maintenance*

- *To what extent are intervention effects maintained among the target population?*
  - **Are there still effects of the intervention in the long term (≥12 months after the start of the intervention)? If yes, what are these effects?**
  - **What is the attrition rate during the intervention period?**
- *Is the intervention continued*
  - **Do the participating organizations still deliver the intervention?**
  - **If not, for how long have they continued the intervention?**
- *Is the intervention institutionalized*
  - **Has sustainability of the intervention been taken into account in any way? E.g. by incorporating/embedding into an existing structure?**
- *To what extent is the continued intervention modified*
  - **Have the participating organizations modified the intervention in any way?**
- *Should all intervention components be maintained*
  - **Are all components maintained? If not, what were the reasons for stopping the concerning components?**
  - **Are there any components that should perhaps in the future no longer be continued? If yes, why?**
- **Are there any other remarks?**
- *Contact information*

**Name** *(Name)*

**Organisation** *(Organisation)*

**Phone number** *(Phone number)*

**E-mail address** *(E-mail address)*

**Intervention website:**

**Other relevant websites:**
